# LHCSR Expression under *HSP70/RBCS2* Promoter as a Strategy to Increase Productivity in Microalgae

**DOI:** 10.3390/ijms19010155

**Published:** 2018-01-05

**Authors:** Federico Perozeni, Giulio Rocco Stella, Matteo Ballottari

**Affiliations:** Dipartimento di Biotecnologie, Università di Verona, Strada Le Grazie 15, 37134 Verona, Italy; federico.perozeni@univr.it (F.P.); giulioste@gmail.com (G.R.S.)

**Keywords:** photosynthesis, photoprotection, microalgae, plant biotechnology, light harvesting, non-photochemical quenching

## Abstract

Microalgae are unicellular photosynthetic organisms considered as potential alternative sources for biomass, biofuels or high value products. However, limited biomass productivity is commonly experienced in their cultivating system despite their high potential. One of the reasons for this limitation is the high thermal dissipation of the light absorbed by the outer layers of the cultures exposed to high light caused by the activation of a photoprotective mechanism called non-photochemical quenching (NPQ). In the model organism for green algae *Chlamydomonas reinhardtii*, NPQ is triggered by pigment binding proteins called light-harvesting-complexes-stress-related (LHCSRs), which are over-accumulated in high light. It was recently reported that biomass productivity can be increased both in microalgae and higher plants by properly tuning NPQ induction. In this work increased light use efficiency is reported by introducing in *C. reinhardtii* a *LHCSR3* gene under the control of *Heat Shock Protein 70*/*RUBISCO small chain 2* promoter in a *npq4 lhcsr1* background, a mutant strain knockout for all *LHCSR* genes. This complementation strategy leads to a low expression of *LHCSR3*, causing a strong reduction of NPQ induction but is still capable of protecting from photodamage at high irradiance, resulting in an improved photosynthetic efficiency and higher biomass accumulation.

## 1. Introduction

Photosynthetic organisms convert light energy into chemical energy to be used for carbon fixation to sustain cellular metabolism. Light harvesting occurs at the level of pigment binding protein complexes called Photosystem I (PSI) and II (PSII) [1,2]. Light energy absorbed by pigments bound to Photosystems causes formation of chlorophyll-excited states and energy transfer to reaction centers inducing charge separation and electron transport across thylakoid membranes. Light energy is thus used to extract electrons from water and transfer them to NADPH. This light dependent electron transport chain involves different electron acceptors and donors among which cofactors bound to PSII and PSI, plastoquinones, the protein complex Cytochrome b6f (Cyt b6f), plastocyanin, ferredoxin and ferredoxin-NADP^+^ reductase enzyme [3,4,5]. Thylakoidal electron transport is coupled to the formation of a proton gradient across thylakoids which is then used to produce ATP by the chloroplast ATPase. Different photosynthetic organisms such as plants or algae must deal with rapid changes in sunlight quality and quantity which can be very dangerous for the photosynthetic apparatus [6,7]. Indeed, the formation of an excess of chlorophyll excited state can lead to saturation of the photosynthetic apparatus, with consequent increased probability of the population of triplet chlorophyll excited states with long lifetime (ms) which makes it possible to transfer energy to oxygen and form reactive oxygen species (ROS) [7]. Photosynthetic organisms have different photoprotective strategies to light changes, among which the thermal dissipation of the singlet chlorophyll excited states not quenched by photochemistry, in a mechanism known as non-photochemical quenching (NPQ) [8]. NPQ activation has been reported to be particularly important for de-excitation of PSII complexes with closed reaction centers, where photochemical reactions cannot properly consume excitation energy of the chlorophylls bound [9]. NPQ activation and in general regulation of photosynthetic reactions can be easily monitored in vivo by chlorophyll fluorescence measurement, a powerful tool to investigate the response of the photosynthetic apparatus to changing environments [10,11,12,13]. NPQ has been reported in all oxygenic photosynthetic organisms [14,15,16,17,18,19], but while in natural conditions it could be important for survival, NPQ activation decreases light use efficiency by opening a dissipative channel of the light energy absorbed. Indeed, recent studies have demonstrated that biotechnological manipulation of crops or microalgae cultures inducing a reduction of NPQ activation or a faster recovery from dissipative state significantly increased photosynthetic efficiency and biomass production [20,21]. Increasing productivity in microalgae is a highly debated topic since microalgae have been proposed as a sustainable alternative for biofuels and food production, since these organisms have high potential photosynthetic efficiency, do no compete for arable land and can be cultivated using nutrient from wastewater [22,23,24,25,26,27]. In the model organism for green algae, *Chlamydomonas reinhardtii*, NPQ is triggered by the pigment-binding subunits LHCSR1 and LHCSR3, two LHC (light harvesting complex)-like Chl a/b-xanthophyll-binding proteins; these subunits have been reported to trigger NPQ induction on the basis of lumenal pH, being able to significantly quench excitation energy present at the level of both PSI and PSII [15,20,28,29,30,31]. Both genes for LHCSR1 and LHCSR3 are transcriptionally regulated causing overaccumulation of these subunits in stress conditions [15,32]. Three genes encode LHCSR proteins in *C. reinhardtii*: two of them are closely related and encode the same product (LHCSR3) while the other one encodes the LHCSR1 subunit, which shows 82% identity to LHCSR3 [15]. All the different genes for LHCSR subunits in *C. reinhardtii* are transcriptionally controlled, depending on light, CO_2_ concentration and calcium signaling [15,32,33] Through random insertional mutagenesis, *npq4* mutant, a knockout mutant for both *LHCSR3.1* and *LHCSR3.2* genes, was obtained, accumulating only LHCSR1 subunit [15], while by TILLING (Targeting Induced Local Lesions IN Genomes) a knockout mutation for *LHCSR1* was added, obtaining the *npq4 lhcsr1* mutant which does not accumulate any LHCSR proteins [28,34]. These mutants revealed that even if both LHCSR subunits are active, LHCSR3 has a predominant role in NPQ induction [15]. Indeed, *npq4* mutant was characterized by a strong NPQ reduction associated with a significant increase in biomass productivity [20] while the *npq4 lhcsr1* showed similar growth rates compared to WT in continuous light conditions [34]. Interestingly, despite a ~60% reduction in NPQ induction, *npq4* was not more photo inhibited compared to WT, while *npq4 lhcsr1* mutant was characterized by the highest production of ROS [20]. These results suggested that it is possible to improve biomass production in *C. reinhardtii* reducing NPQ activation if a basal level of photoprotection is ensured by accumulation of some LHCSR subunits, such as LHCSR1 in the case of *npq4* mutant [20]. In this work, a different strategy to obtain strains with reduced NPQ and increased photosynthetic efficiency was addressed, by complementation of *npq4 lhcsr1* mutant with *LHCSR3* gene under control of a promoter obtained by fusion of *Heat Shock Protein (HSP)70* and *RUBISCO small subunit (RBCS) 2* promoters [35]: *LHCSR3* gene controlled by *HSP70/RBCS2* promoter is not strongly induced in high light resulting in a low expression of this gene and reduced NPQ compared to WT. This strategy could be indeed required for the biotechnological manipulation of other microalgae species inducing a basal accumulation of LHCSR subunits in mutants with NPQ phenotypes.

## 2. Results

### 2.1. Complementation of npq4 lhcsr1 Strain

**Figure 1:**
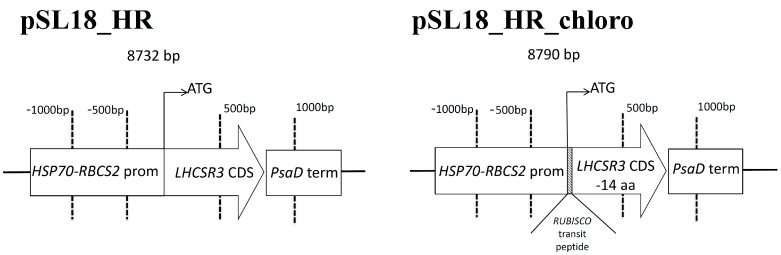
Schematic maps of the vectors used for *npq4 lhcsr1* transformation. *LHCSR3* coding sequence (*LHCSR3* CDS) was cloned in pSL18 vector under the control of promoter obtained by fusion of *HSP70* and *RBCS2* promoter (pSL18_HR). In the case of pSL18_hR_chloro, *LHCSR3* coding sequence was truncated at the 5′ in order to remove the codons coding for the first 14 aminoacids of the protein: chloroplast transit peptide was thus substituted with *RUBISCO* (rbcs) small subunit transit peptide. For both vectors PsaD terminator sequence was used as terminator.

*C. reinhardtii* strains with a constitutive expression of *LHCSR3* gene were obtained by complementation of *npq4 lhcr1* mutant with *LHCSR3* coding sequence under control of a promoter obtained fusing promoters of *HSP70* and *RBCS2* genes as previously reported [35] (Figure 1). In particular, two vectors were used for complementation differing in the presence of transit peptides: pSL18_HR contained LHCSR3 whole coding sequence, including its putative transit peptide, while in pSL18_HR_chloro LHCSR3 coding sequence was deleted of the first 42 nucleotides, corresponding to the putative 14 amino acids chloroplast transit peptide, according to ChloroP prediction [36], which were replaced by the RUBISCO small subunit transit peptide [37].

**Figure 2:**
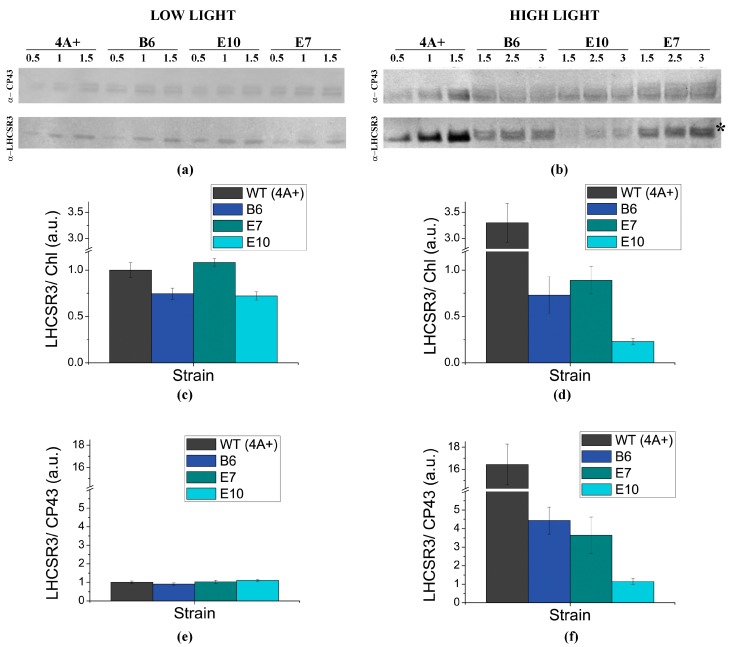
Immunoblot analysis of LHCSR3 accumulation in transformed lines compared to WT (4A+) strain. (**a**,**b**): immunoblot analysis for strains grown in low light (**a**) or high light (**b**) using specific antibodies recognizing LHCSR3 and CP43 as a control for protein loading. Proteins extracted from WT and transformed lines were loaded at three different dilutions as reported in the figure. The phosphorylated form of LHCSR3 is indicated by *; (**c**–**f**): accumulation of LHCSR3 in low light and high light treated cells determined by densitometric analysis of filters reported in (**a**,**b**) respectively. LHCSR3 content was normalized to the chlorophyll (Chl) content (**c**,**d**) or to CP43 content (**e**,**f**). The amount of LHCSR3/Chl and LHCSR3/CP43 ratios were then normalized the ratios detected in WT cells grown in low light conditions. In the case of WT grown in high light conditions, LHCS3/Chl and LHCSR/CP43 ratios were normalized to the levels detected for WT cells grown in low light according to results reported in Figure S2. Values in (c, d, e, f) are indicated as arbitrary units (a.u.) with standard deviation reported as error bar (*n* = 3).

Transformed lines surviving antibiotic selection (Figure S1A) were grown in high light conditions and screened for NPQ induction compared to the background strain. NPQ screening allowed the identification five lines transformed with pSL18_HR and one line transformed with pSL18_HR_chloro with an increased NPQ compared to *npq4 lhcsr1*, suggesting the accumulation of a functional LHCSR3 protein (Figure S1B). LHCSR3 protein accumulation was then investigated by immunoblotting, confirming the presence of LHCSR3 protein in transformed lines with an increased NPQ phenotype compared to *npq1 lhcsr1* mutant (Figure S1C). Three of these lines were then selected for the following experiments: two lines transformed with pSL18_HR (lines E7 and E10) and one line transformed with pSL18_HR_cloro (line B6). LHCSR3 level in these complemented lines was quantified by immunoblotting in cells grown at low light (Figure 2A) or high light (Figure 2B) conditions, using the PSII subunit CP43 as control for loading. As reported in Figure 2A, in low light all transformed lines showed a LHCSR3 accumulation on chlorophyll basis (Figure 2C) or normalized to CP43 content (Figure 2E) comparable to the case of WT cells grown in the same conditions. In high light conditions, WT cells showed a strong increase of LHCSR3 accumulation compared to cells grown in low light both on a chlorophyll basis or upon normalization to CP43 content, leading to a strong increase of LHCSR3/PSII ratio, as previously reported [38,39] (Figure 2, Figure S2). Differently, transformed lines showed only a partial increase of LHCSR3 accumulation on a chlorophyll basis (Figure 2D) or per PSII (Figure 2F) in high light. In both low and high light conditions, E10 line showed a protein level slightly lower compared to the other transformed lines, which could be ascribable to a position effect. It is worth to note that in transformed lines, the phosphorylated form of LHCSR3, migrating at slightly higher apparent MW compared to the un-phosphorylated form [36,40], was particularly evident in high light-grown transformed lines, in agreement with the higher activity of the STT7 kinase in high light in mutants with reduced NPQ induction [41].

### 2.2. Pigment Composition of Transformed Lines

Pigment composition of WT and transformed lines growing in low light or high light was investigated by HPLC analysis (Table 1) when cells reached the exponential growth phase. Chlorophyll content per cell was reduced in transformed lines, both in low light and high light, compared to WT. In addition, an increased Chl a/Chl b ratio and carotenoid content were measured in transformed lines in both light conditions. Chl/Car ratio was further reduced in transformed lines grown in high light. It is interesting to note that the Chl/Car ratio of B6, E7 and E10 strains grown in low light is similar to the Chl/Car ratio of WT grown in high light, where the Chl/Car of transformed lines is further decreased. Intermediate values of Chl a/b ratio between WT and transformed lines were measured for the background strain *npq4 lhcsr1* grown in low light, while Chl a/b ratio in high light, Chl/Car ratio and Chl content per cell both in low light and high light were similar between WT and *npq lhcsr1* (Table S1), suggesting the different pigment binding properties observed in transformed lines are independent from the genetic background but rather related to an adaptation process to the growing conditions likely related to reduced NPQ induction observed in these strains with consequent increased excitation pressure at the level of PSII. As reported in Supplemental data Table S2 the average cell size was not significantly different for transformed lines compared to WT, neither in low light nor high light conditions, suggesting that the reduced chlorophyll content per cell observed in transformed lines is likely dependent on adaptation processes occurring at the level of the chloroplast.

### 2.3. NPQ Induction and LHCSR3 Accumulation

**Figure 3:**
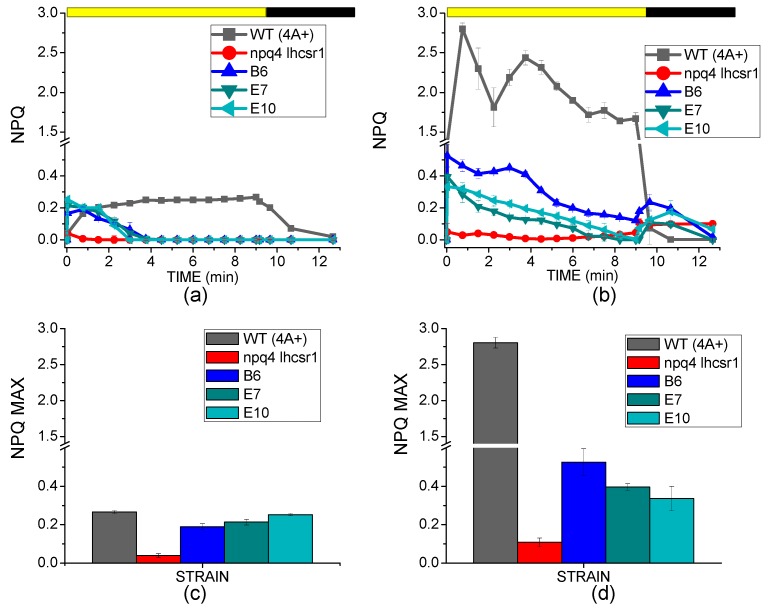
NPQ kinetics and NPQ maximum induction. (**a**,**b**): NPQ kinetics measured in cells grown in low light (**a**) or high light (**b**) upon actinic light illumination (1200 μmol m^−2^·s^−1^), and dark recovery. Yellow bar indicates the illumination period and the dark bar indicates the dark recovery; (**c**,**d**): maximum NPQ values detected for WT, transformed lines and their background *npq4 lhcsr1* grown in low light (**c**) or high light (**d**) with standard deviation reported as error bar (*n* = 3).

NPQ induction kinetics of WT, *npq4 lhcsr1* mutant and complemented lines are reported in Figure 3 for cells grown in low light or high light. Low light-acclimated WT cells were characterized by a lower NPQ induction compared to high light cells (Figure 3A,B) as previously reported, because of light dependent accumulation of LHCSR3 in *C. reinhardtii* [15,38]. Differently, *npq4 lhcsr1* mutant did not show any NPQ induction neither in low or high light, due to the absence of LHCSR subunits in this strain [20,28,34]. Complemented lines B6, E7 and E10 were characterized by an initial rise of NPQ induction during the first minute of measurement, followed by a decrease even if actinic light was still turned on. In low light, the maximum NPQ induction observed for WT and transformed lines was similar (Figure 3C), but while WT reached the maximum NPQ value at the end of the illumination period, complemented lines reached maximum NPQ induction after 30 s followed by a strong decrease in NPQ. A similar behavior was also observed in high light, with the maximum NPQ level observed in transformed lines only at the beginning of the measurement. Maximum NPQ level observed in the transformed line was thus much lower compared to WT (Figure 3D); the dependency of NPQ maximum induction vs. accumulation of LHCSR3 protein is reported in Supplemental data Figure S3, suggesting a linear correlation between the amount of LHCSR3 per chlorophyll and the maximum NPQ value observed, which is in agreement with previous reports [20,38]. Moreover, NPQ in complemented lines decreases after few minutes in low light or seconds in high light; this result can be related to light-dependent activation of the Calvin cycle and de-saturation of photosynthetic electron chain. In order to further elucidate this point, the accumulation of the different photosynthetic subunits was investigated by immunoblotting.

### 2.4. Composition of Photosynthetic Subunits

**Figure 4:**
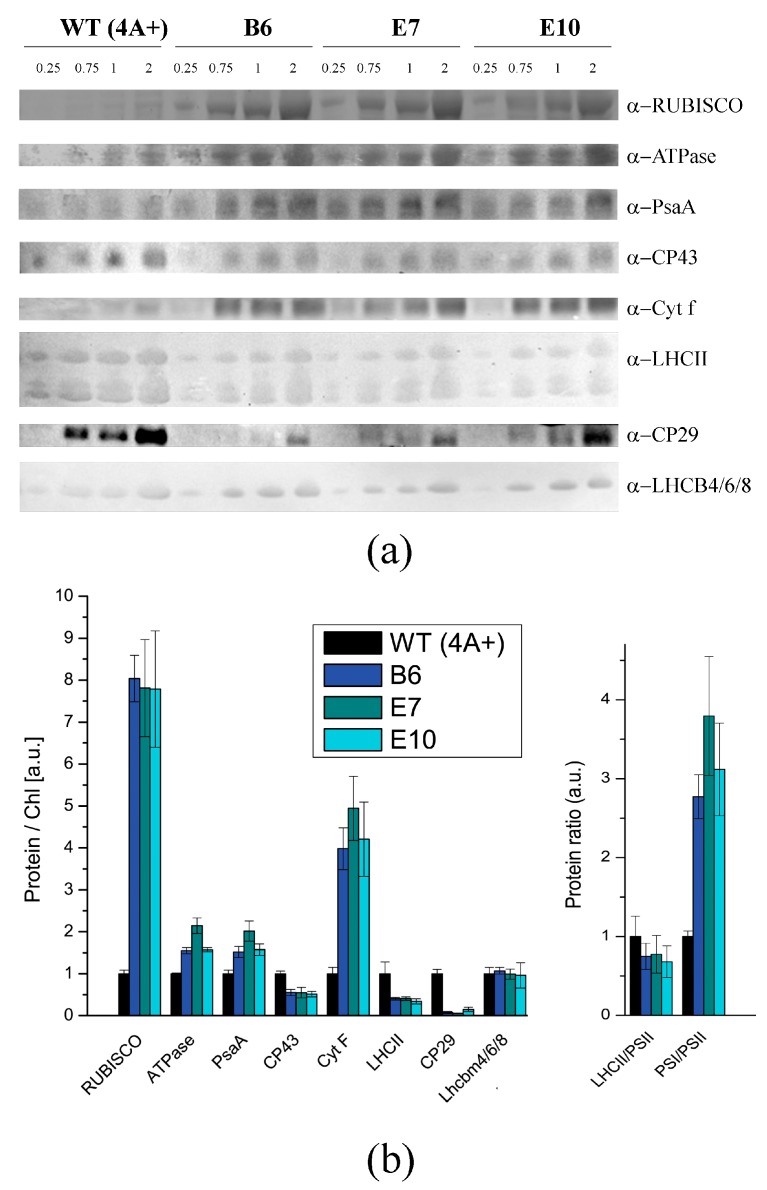
Immunoblot analysis of photosynthetic subunit accumulation. (**a**): immunoblot analysis performed on WT and transformed lines proteins extract using specific antibodies for RUBISCO, ATPase β-subunit, PsaA, CP43, Cyt f, LHCII, CP29 and LHCBM4/6/8 subunits. Micrograms of chlorophylls loaded in each lane are reported on the top. (**b**): Immunoblot signals reported in panel A were analyzed by densitometry to determine the relative protein abundance. Each protein level was normalized to the WT protein level. LHCII/PSII and PSI/PSII ratio are also reported. Standard deviation is indicated as error bars (*n* = 4).

Accumulation of the major photosynthetic complexes was investigated by semiquantitative immunoblotting with specific antibodies targeting proteins representative of PSI, PSII, Cytb6f, ATPase and RUBISCO. In addition, immunoblotting analysis were performed using antibodies recognizing specifically the PSII antenna proteins CP29, LHCBM4/6/8 and an antibody recognizing all the different LHCII subunits (Figure 4) [42]. Transformed lines were characterized by a higher accumulation of RUBISCO compared to WT, either on a chlorophyll (Figure 4) or cell (Figure S4) basis. Transformed lines were also characterized by a strong increase of Cyt f, α-ATPase, and PsaA on a chlorophyll basis accompanied by a reduced accumulation of CP43 compared to WT, resulting in an increased PSI/PSII, Cytb6f/PSII and ATPase/PSII ratio. Interestingly, on a cell basis, the accumulation of PsaA and ATPase was similar in WT and transformed lines, while CP43 was strongly reduced and Cyt f was increased in transformed lines (Figure S4). These results suggest that the reduced NPQ activation observed in transformed lines causes a specific reduction of PSII accumulation on a cell basis, likely as a consequence of the increased excitation pressure due to reduced NPQ. In the case of PSII antenna proteins, on a chlorophyll basis CP29 and LHCII subunits decreased compared to WT, while LHCBM4/6/8 level was not significantly different. Considering the level of CP43, the stoichiometry LHCII/PSII was similar in WT and transformed lines, with the exception of LHCBM4/6/8 subunits which were increased per PSII. It is important to note that LHCBM4/6/8 was recently reported to have a minor role in light harvesting at the level of PSII but was rather involved in state transitions and light harvesting at the level of PSI [42]. Increased PSI/PSII ratio measured in transformed lines is consistent with the increase in Chl a/b ratio reported in Table 1, since Chl a/b ratio in the case of PSI is higher than the Chl a/b ratio observed in the case of PSII. The results obtained for transformed lines suggest an increased possible consumption of ATP and NADPH produced by light phase by carbon fixation, and at the same time a relative increase of electron acceptors downstream of PSII, as Cytb6f and PSI. Different accumulation of photosynthetic subunits likely allows dealing more efficiently with the increased excitation pressure on PSII due to reduced NPQ. Similar immunoblotting analyses were also performed comparing WT and *npq4 lhcsr1* to evaluate possible differences related to the background strain (Figure S5); no differences were observed between WT and *npq4 lhcsr1*, with the only exception of a more evident reduction of LHCII content in *npq4 lhcsr1*. These results confirm that the different accumulation of photosynthetic subunits observed in transformed lines are related to the expression of the LHCSR3 subunit and not to their genetic background.

### 2.5. Light Dependent Oxygen Evolution vs. Photoprotection

**Figure 5:**
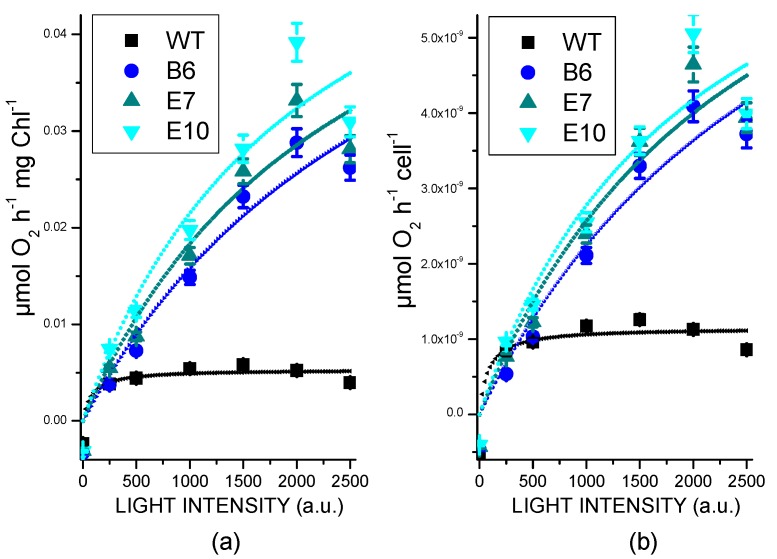
Light saturation curves of photosynthetic oxygen evolution obtained from WT (4A+) and transformed lines. Data are reported on chlorophyll basis (**a**) or on a cell basis (**b**). Standard deviations are reported as error bars (*n* = 3).

Photosynthetic activity of transformed lines compared to WT was evaluated measuring light-dependent oxygen evolution curves at different actinic light intensities (Figure 5). Transformed lines showed higher Pmax than WT, demonstrating an increasing light use efficiency for these strains. These data are consistent with improved light-dependent electron transport across thylakoids in transformed lines. Differently, dark respiration per cell was similar in transformed lines compared to WT.

**Figure 6:**
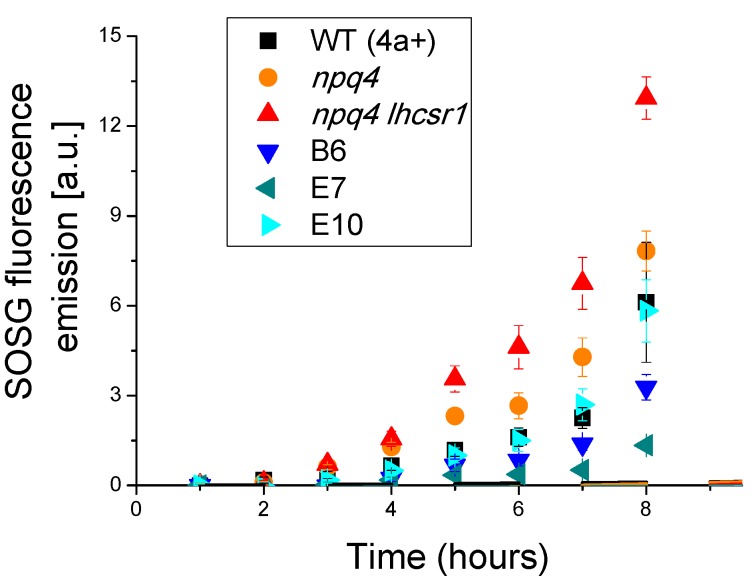
Singlet oxygen production for WT and transformed lines. Singlet oxygen production was estimated from fluorescence emission of Singlet Oxygen Sensor Green (SOSG) probe. Data reported are the mean values of three independent biological replicates with standard deviations indicated as error bars.

Increased photosynthetic activity of transformed lines characterized by reduced NPQ activation opens the question about the photoprotective properties of these strains. Singlet oxygen formation was thus followed in vivo upon exposure of cells to a strong red light by using a fluorescent probe called Singlet Oxygen Sensor Green [43], of which the fluorescence emission is linearly dependent on the concentration of singlet oxygen [44]. As reported in Figure 6, transformed lines were characterized by a similar or even less singlet oxygen production compared to WT, while their background strain *npq4 lhcsr1* was the strain with the highest singlet oxygen production. This data suggests that, as previously observed in the case of LHCSR1 accumulated at low level in *npq4* mutant, photoprotection provided by the small amount of LHCSR3 accumulated in WT was sufficient to preserve the photosynthetic apparatus from photoinhibition.

### 2.6. Biomass Productivity

**Figure 7:**
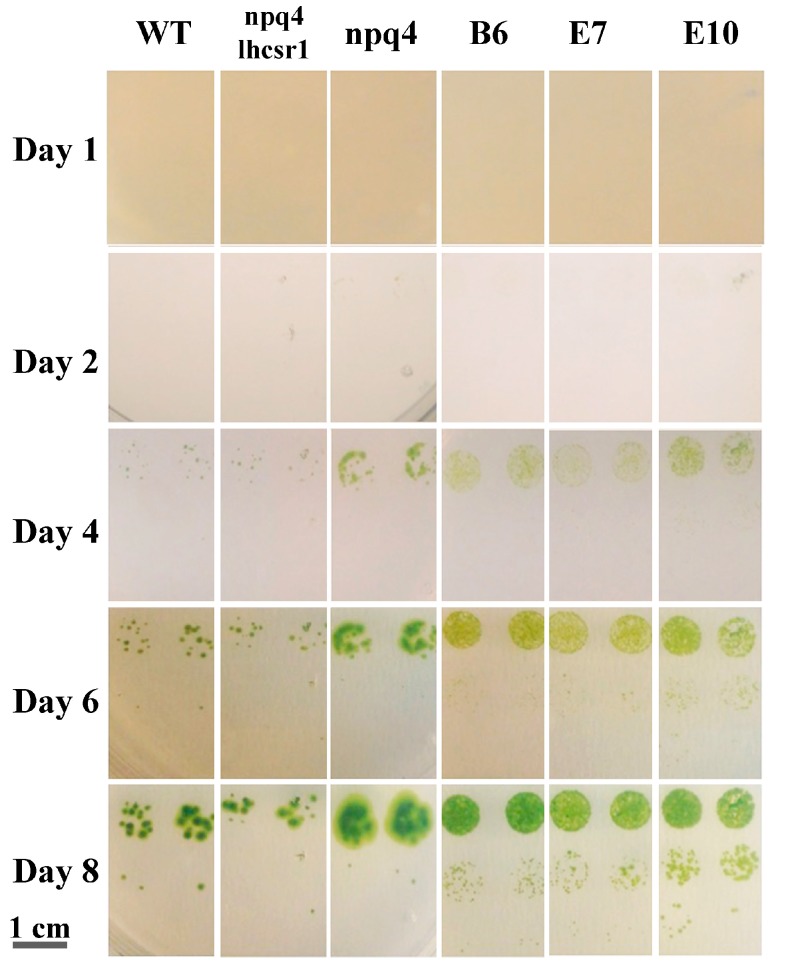
Spot test for WT and transformed lines. Each genotype was spotted on solid minimum medium with three different cell amount (10^2^,10^3^ and 10^4^ cells). Scale bar is reported on bottom left.

Transformed lines with *LHCSR3* gene under control of the *HSP70/RBCS2* promoter were characterized by reduced NPQ induction compared to WT and increased photosynthetic activity (Figure 5) without suffering from photoinhibition (Figure 6). In order to prove a possible increased biomass productivity of transformed strain, growth performances in solid medium in high light was tested by spotting different cell concentrations for each genotype on HS medium (Figure 7A) and comparing transformed lines to WT, *npq4* and *npq4 lhsr1* strains. As reported in Figure 7, *npq4* and transformed lines were characterized by a faster and increased growth compared to WT, while the lowest growth performance was detected for *npq4 lhcsr1* strain.

**Figure 8:**
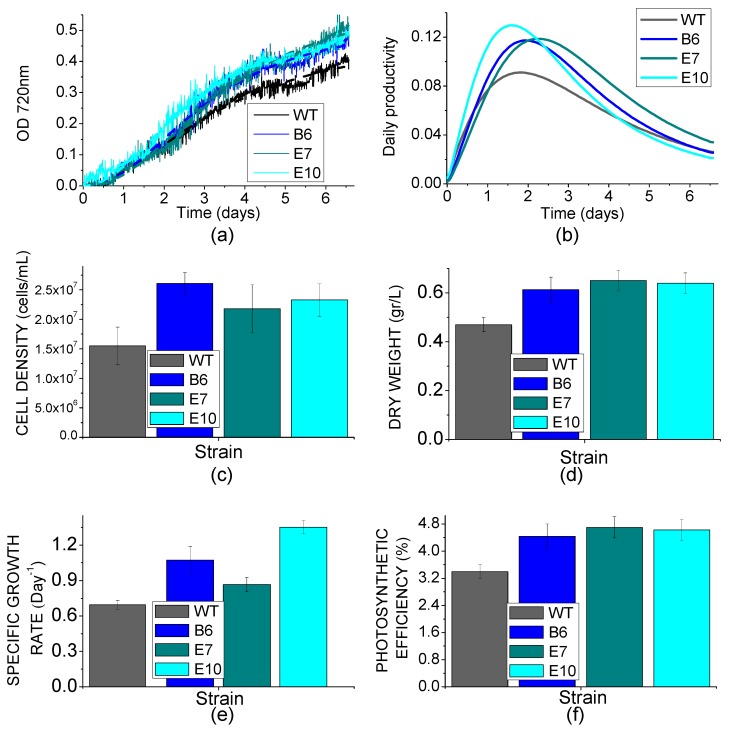
Biomass productivity in photobioreactors. (**a**): growth curves of WT and transformed lines obtained by measuring the optical density (O.D.) at 730 nm.. Growth curves were fitted with an exponential growth function; (**b**): daily productivity estimated from the first derivate of the fitting curves reported in (**a**); (**c**): cell density obtained at the end of the growth curve; (**d**): dry weight obtained at the end of the growth curve; (**e**): specific growth rate (μ) for WT and transformed lines; (**f**): photosynthetic efficiency of WT and transformed lines calculated from dry weight obtained at the end of growth curve. Mean value of three independent measurements are reported. Standard deviation are reported as error bars (*n* = 3).

Biomass productivity was then investigated in liquid medium by growing transformed lines in small scale photobioreactors (Multicultivator MC-1000-OD), at 400 μmol m^-2^·s^-1^. As reported in Figure 8A, transformed lines grew faster than WT reaching higher cell density at the end of the growth curve (Figure 8B). Daily productivity was then estimated from the first derivate of the sigmoidal curves used to fit growth curves (Figure 8C), showing a ~35% higher maximal daily productivity in transformed lines compared to WT. Accordingly, dry weight of biomass collected at the end of the growth was increased in transformed lines by ~34% compared to WT (Figure 8D). Consistently, the specific growth rate (μ) calculated for transformed lines was increased by ~18% compared to WT (Figure 8E). It is worth to note that the specific growth rates reported in Figure 8D are consistent with previous growth rates reported for *C. reinhardtii* [45,46,47,48]. Photosynthetic efficiency of WT and transformed cultures were then calculated considering the total amount of photons received by the cultures and the energy stored as dry weight biomass at the end of growth curves; WT cells exhibited a photosynthetic efficiency of 3.4%, while transformed lines scored photosynthetic efficiencies of 4.4–4.7% (Figure 8F). While growth kinetics and biomass accumulation of *npq4 lhcsr1* was reported to be similar or lower compared to WT [34], complementation of *npq4 lhcsr1* mutant with *LHCSR3* gene under a constitutive promoter caused a significant increase of photosynthetic efficiency and biomass accumulation compared to WT.

## 3. Discussion

NPQ is a feedback photoprotective mechanism triggered in oxygenic photosynthetic organisms to decrease the risk of photoinhibition and cell death [7]. NPQ quenches chlorophyll singlet excited states reducing the probability of chlorophyll triplet formation and ROS production [49]. However, excitation energy quenched by NPQ is unavoidably loss for photochemical reactions, causing a decrease in photosynthetic efficiency [20,50]. Previous reports on the *npq4* mutant demonstrated that a reduced NPQ activation in the absence of LHCSR3 protein caused an increase of biomass accumulation compared to WT due to increased photosynthetic efficiency [20]; *npq4* mutant was reported to produce a similar level of singlet oxygen upon exposure to strong light compared to WT, despite strong reduction of NPQ, while the double mutant *npq4 lhcsr1* was more photo-inhibited than WT and *npq4* due to complete suppression of light-dependent NPQ activation [20]. It is worth to mention that a recent paper reported a similar biomass accumulation and CO_2_ fixation upon continuous cultivation of *npq4* mutant and WT, while previous work reported an even stronger light susceptibility of *npq4* strain [15,41,51]. Contrasting results reported in the literature about the performances of *npq4* mutant are likely related to the different environmental conditions adopted for cultivation. In particular, the need for an efficient NPQ activation could be attenuated in the presence of high CO_2_ content, resulting in a more active Calvin–Benson cycle as a sink for electrons transported during the light phase of photosynthesis. Moreover, the residual NPQ observed in *npq4* mutant depends on LHCSR1 subunit, the accumulation of which is strictly controlled at the transcriptional level by high light and high CO_2_ content [32]. Residual NPQ observed in *npq4* mutant is indeed related to LHCSR1 overexpression to partially counterbalance LHCSR3 absence and to ensure the minimum level of photoprotection required to sustain growth in high light condition [20]. In this work, we demonstrate a different strategy to increase photosynthetic efficiency by reducing NPQ, consisting of low expression of *LHCSR3* gene in absence of LHCSR1. Complementation of *npq4 lhcsr1* mutant with LHCSR3 genes under *HSP70/RBCS2* promoter (Figure 1) indeed resulted into accumulation of LHCSR3 protein similar to WT in LL conditions, but not in HL, where transformed lines were characterized by a strong reduction of LHCSR3, due to the strong light dependent transcriptional activation of the endogenous LHCSR3 gene in the WT (Figure 4). As reported in Figure 6, transformed lines with low NPQ induction were characterized by similar or even lower singlet oxygen production compared to WT, demonstrating that the excitation energy not quenched by NPQ could be successfully managed by the photosynthetic apparatus with, therefore, a 30% increase of biomass accumulation in transformed lines (Figure 8). Expression of LHCSR3 at low levels is thus a sufficient condition in *C. reinhardtii* to ensure enough photoprotection at least in the growth conditions herein tested, allowing for a higher photosynthetic efficiency (Figure 5 and Figure 8F), because of reduced NPQ (Figure 3). We cannot exclude that more stress conditions, at higher irradiances, could impair the productivity of transformed lines, even if the singlet oxygen production of these strains illuminated with a strong light was similar or even lower than WT; additional experiments are required to elucidate the potential limits of this strategy to increase biomass productivity. It is interesting to note that NPQ induction curves of transformed lines were markedly different compared to WT, since the maximum NPQ level was observed only in the first minutes or seconds after exposure to actinic light, followed by a strong decrease. This result is likely related to light-dependent activation of Calvin cycle enzymes which consume NADPH and ATP, reducing the excitation pressure on PSII, when the CO_2_ concentration is sufficient to provide a sink for photosynthetic light phase products. This hypothesis is sustained by the strong increase in RUBISCO enzyme observed in transformed lines compared to WT both on a chlorophyll or cell basis (Figure 4, Supplemental data Figure S4), suggesting that the reduction in NPQ observed in transformed lines causes an enhanced Calvin–Benson cycle and a more general re-organization of the photosynthetic apparatus. Decrease in chlorophyll content was observed both in transformed lines coupled with increased Chl a/Chl b ratio and related to increased PSI/PSII ratio. Reduction of PSII content on a cell basis is likely the main reason for the reduced chlorophyll content observed in the case of transformed lines compared to WT (Table 1). The decreased PSII content on a cell basis is likely not related to PSII damage, since the oxygen evolution curves of transformed lines are characterized by a higher Pmax compared to WT, while in case of damaged PSII, the opposite would be expected. Rather, relative increase of PSI and Cytb6f compared to PSII content in transformed lines suggests a more efficient use of electrons coming from water splitting at the level of PSII. This adaptation strategy is likely adopted by transformed lines in order to improve oxidation of Q_A_ at the level of PSII reducing the risk of photoinhibition even in the presence of a strongly reduced NPQ activation. The higher resistance of transformed lines to ROS production can thus be explained as a combination of improved electron transport across thylakoid, increased electron sink due to relative increase of Calvin–Benson enzymes and reduced Chl/Car ratio (Table 1), with carotenoids being important photoprotective agents inducing chlorophyll triplets quenching and ROS scavenging [7]. In this scenario, transformed lines can better face high light stress and use light energy thanks to a boosted photosynthetic electron transport and carbon fixation machinery; the sum of these elements allows for improved light use efficiency and, thus, biomass accumulation. It is interesting to note that the different compositions of photosynthetic subunits in transformed lines are in line with what has been previously observed for *npq4* mutant compared to WT and for WT cells adapted to high light compared to low light grown cells. These results suggest that the reduction of NPQ observed in transformed lines induces cellular acclimation with a similar mechanism compared to WT cells exposed to increased irradiances due to the increased excitation pressure on photosystems when thermal dissipation of singlet chlorophyll excited states is reduced. The comparison between transformed lines with *LHCSR3* gene under *HSP70/RBCS2* promoter and their background, *npq4 lhcsr1* mutant, demonstrate how the biochemical changes observed in transformed lines are not dependent on their background; the presence of LHCSR3 ensures a minimal level of photoprotection which allows the chloroplast to adapt the photosynthetic apparatus in order to exploit, as much as possible, the unquenched excitation energy. In addition, since LHCSR subunits have been previously reported to interact with both PSII and PSI [52,53,54], it cannot be excluded that LHCSR1 or LHCSR3 in *C. reinhardtii* could be required for this light-dependent adaptation process of the thylakoid membranes by controlling PSII and/or PSI stabilization in high light-treated cells; this possible hypothesis requires additional work to be elucidated.

In summary, the results herein reported provide additional information on the relationship between NPQ and productivity in *C. reinhardtii*, suggesting that a possible strategy to improve light use efficiency and biomass productivity is controlling the expression of *LHCSR3* gene by a promoter which is not strongly light inducible as in the case of its endogenous promoter, causing a strong reduction of NPQ induction in high light-treated cells. This strategy, herein reported for the model organism *C. reinhardtii*, could be transferred to other algal species, such as those with high commercial interest; in this case, knockout of potential high light-upregulated *LHCSR*-like genes could be obtained by genome editing or random mutagenesis, followed by complementation of LHCSR gene(s) under the control of a constitutive promoter. It is worth to mention that the relevance of NPQ induction in the natural environment is likely high, considering the ubiquitous identification of NPQ activation among oxygenic photosynthetic organisms. However, in artificial controlled conditions, as those experienced by microalgae in photobioreactors, tuning of NPQ can be a powerful tool to increase biomass productivity.

## 4. Materials and Methods

### 4.1. Chlamydomonas Reinhardtii Transformation

*LHCSR3* coding sequence was obtained by PCR amplification of cDNA obtained from RNA extracted from high light treated 4A+ cells [36]. LHCSR3 coding sequence was then cloned in two vectors called pSL18_HR and pSL18_HR_chloro. Both vectors are derived from PSL18 vector [55] controlling the inserted sequence with a promoter obtained by fusion of heat shock protein 70 e RUBISCO small subunit promoters [35]. In the case of PSL18_HR_chloro the inserted LHSR3 coding sequence was deleted of the first 42 bases to remove the endogenous transit peptides, which was substituted by the transit peptide of RUBISCO small subunit [56]. *npq4 lhcsr1* strain [28] was transformed by electroporation as described in [55]. Selection of transformed colonies were performed in solid medium in presence of paromomycin. 

### 4.2. Chlamydomonas Reinhardtii Cultivation

*C. reinhardtii* cells of strains 4A+, CW15, *npq4*, *npq4 lhcsr1* [15,20,57] and transformed lines were grown in minimum medium (HS medium) in flasks in control conditions (70 μmol m^−2^·s^−1^; photoperiod of 16/8 h of light/dark). Growth analysis in liquid medium was performed in a small scale photobioreactors provided by Multi-Cultivator MC 1000 (Photon System Instruments, Brno, Czech Republic) as described in [20]. The samples were grown for one day at 70 μmol m^−2^·s^−1^, and then at 400 μmol m^−2^·s^−1^. Each experiment started with 5 × 10^5^ cells/mL in minimal medium (HS medium) enriched with NaHCO_3_ (0.5 g/L). Cell density in the Multi-Cultivator MC 1000 tubes was automatically monitored every ten minutes by measuring cell dependent scattering at 730 nm. The samplings for pigment analysis, immunoblotting, fluorescence and photosynthetic measurements were carried out on cultures in exponential growth phase. At the end of the exponential phase of growth, the cells were counted at the microscope using an improved Neubauer hematocytometer. Specific growth rates were calculated from liner regression of the exponential growth phase in logarithmic scale as described in [48]. Cell dry weight was measure upon drying biomass for 4 days at 60 °C. Spot test was performed spotting cells grown at 70 μmol m^−2^·s^−1^ at mid-exponential phase. In particular 10^2^, 10^3^ and 10^4^ cells were spotted HS medium with 1% agar added; plates were then exposed to high light (400 μmol m^−2^·s^−1^) for eight days.

### 4.3. Chlorophyll Fluorescence and Oxygen Evolution Measurement

A video imaging system designed for acquiring fluorescence (FluorCam 800MF by Photon System Instruments, Drasov, Czech Republic) was used for screening transformed lines for a phenotype of increased NPQ compared to their background *npq4 lhcsr1*. NPQ was calculated as Fm/Fm’ − 1 according to [8], where Fm is the maximum fluorescence emitted upon exposure to a saturating flash (3000 μmol m^−2^·s^−1^) of dark adapted cells, while Fm’ is the maximum fluorescence emitted upon exposure to a saturating flash of cells exposed to actinic light (1200 μmol m^−2^·s^−1^). NPQ measurements were also performed using a PAM-110 fluorometer with a saturating light at 5000-μmol photons m^−2^·s^−1^ and actinic light of 1500 μmol photons m^−2^·s^−1^. Before measurements, cells were dark-adapted under stirring for at least 60 min at room temperature. Photosynthetic O_2_ production was measured on whole cells at 25 °C using a Clark electrode for the liquid phase oxygen measurements (Oxy-Lab, Hansatech Instruments Ltd., Norfolk, UK) [20].

### 4.4. Pigment Analysis

Pigments analysis were performed upon pigments extraction in 80% acetone and HPLC analysis as previously described [58].

### 4.5. SDS-PAGE Analysis, Immunoblot Assays and Western Blotting Quantifications

Protein extracted from whole cells were analyzed by SDS-PAGE electrophoresis on a 15% acrylamide gel with Tris-Tricine buffer system [59]. Immunoblot assays with antibodies against different polypeptides were performed as previously described [20,42].

### 4.6. Singlet Oxygen Production

Singlet oxygen production was measured in vivo by exposing cells to a high red light (1500 μmol m^−2^·s^−1^) using Single Oxygen Sensor Green fluorescent probe [43] as described in [20].

## Figures and Tables

**Table 1:** Pigments analysis of transformed lines compared to WT. Chlorophyll content per cell, Chl a/b ratio, and Chl/Car ratio are reported with standard deviation (*n* = 4).

		Chl/Cell	s.d.	Chl a/b	s.d.	Chl/Car	s.d.
low light	WT	2.16 × 10^−6^	1.99 × 10^−7^	1.86	0.01	3.93	0.02
	B6	1.42 × 10^−6^	1.26 × 10^−7^	3.22	0.01	2.46	0.01
	E7	1.40 × 10^−6^	2.69 × 10^−7^	3.24	0.03	2.50	0.02
	E10	1.29 × 10^−6^	7.70 × 10^−8^	3.24	0.10	2.49	0.06
high light	WT	7.29 × 10^−7^	1.29 × 10^−8^	1.49	0.04	2.48	0.20
	B6	3.87 × 10^−7^	7.29 × 10^−9^	2.43	0.07	1.39	0.06
	E7	4.38 × 10^−7^	1.09 × 10^−8^	2.53	0.03	1.15	0.03
	E10	4.17 × 10^−7^	9.63 × 10^−9^	2.59	0.05	1.07	0.03

## Supplementary Materials

Supplementary materials can be found at www.mdpi.com/1422-0067/19/1/155/s1.

## Abbreviations

NPQ	Non Photochemical Quenching
PSI/II	Photosystem I, II
LHC	Light Harvesting Complex
LHCSR	Light Harvesting Complex Stress Related
LHCII	Light Harvesting Complex II
HSP	Heat Shock Protein
RBCS2	Ribulose-1,5-bisphosphate Carboxylase/Oxygenase Small Subunit
Chl	Chlorophyll
Car	Carotenoids
