# Supplementary material for: LHCSR Expression under HSP70/RBCS2 Promoter as a Strategy to Increase Productivity in Microalgae
Source: Int J Mol Sci. 2018 Jan 5;19(1):155. doi: 10.3390/ijms19010155 (PMC5796104; doi:10.3390/ijms19010155)

LHCSR expression under HSP70/RBCS2 promoter as a strategy to increase productivity in microalgae

Federico Perozeni [1], Giulio Rocco Stella [1] and Matteo Ballottari [1,*]

[1] 1 Dipartimento di Biotecnologie, Università di Verona, Strada Le Grazie 15, 37134 Verona, Italy
[*] Correspondence: matteo.ballottari@univr.it; Tel.: +39-0.458027807

SUPPLEMENTAL DATA

Table S1: Pigments analysis of npq4 lhcsr1 compared to WT. Chlorophyll content per cell, Chl a/b ratio, and CHl/Car ratio are reported with standard deviation (n=4).

		Chl/cell	*s.d.*	Chl a/b	*s.d.*	Chl/Car	s.d.
low light	**WT**	2.16E-06	*1.99E-07*	1.86	*0.01*	3.93	0.02
	**npq4 lhcsr1**	2.02E-06	*1.23E-07*	2.37	*0.01*	3.86	0.01
high light	**WT**	7.29E-07	3.29E-08	1.49	0.04	2.48	0.20
	**npq4 lhcsr1**	6.87E-07	4.28E-08	1.55	0.07	2.05	0.06

Table S2: Average cell diameter of WT and transformed lines. Average diameter was calculated from cells grown in low light or high light conditions when cells were at the end of their exponential growth. Standard deviation is reported (n=40).

	**Average diameter (μm)**			
	**Low light**	*s.d.*	**High light**	*s.d.*
**WT (4a+)**	8.15	*1.91*	7.98	*2.47*
**B6**	6.58	*1.53*	10.57	*2.12*
**E7**	6.98	*1.55*	9.44	*2.81*
**E10**	6.80	*1.26*	9.13	*2.29*

Figure S1: Screening of transformed lines expressing LHCSR3 protein in *npq4 lhcsr1* background. (a): antibiotic selection in presence of paromomycin compared to survival test (no antibiotic added), and negative control (ctrl), where in the latter npq4 lhcsr1 cells not transformed were plated. (b): maximum NPQ induction observed in transformed lines upon high light adaptation for 5 five days compared to their background npq4 lhcsr1. Strains accumulating both LHCSR1 and LHCSR3 (CW15 and 4A+) or accumulating only LHCSR1 (*npq4*) are also reported for comparison. (c): LHCSR3 accumulation analyzed by immunoblotting reaction.

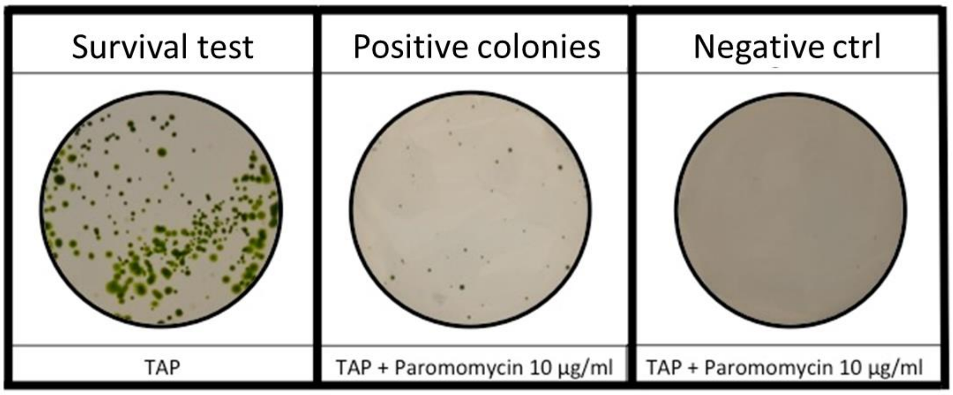

(a)

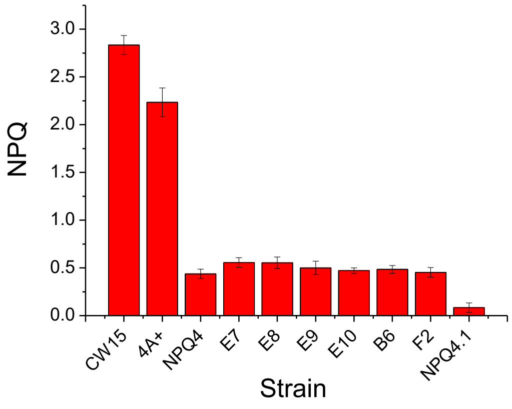

(b)

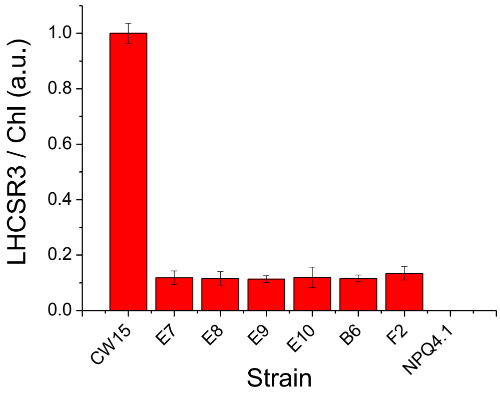

(c)

Figure S2: example of immunoblot analysis on protein extracts from WT and one transformed line grown in low light or high light loaded on the same filter. Immunoblotting was performed using specific antibodies recognizing LHCSR3 and CP43; 1,5 μg of chlorophylls was loaded for each lane.

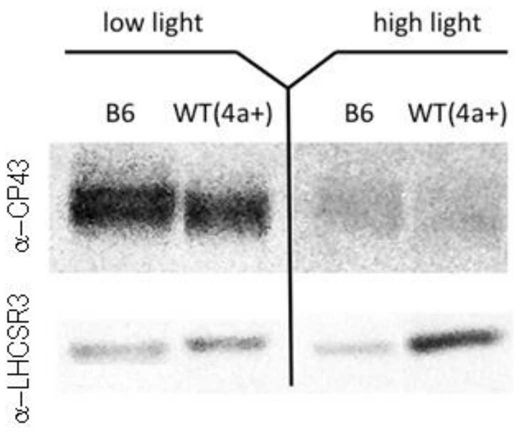

Figure S3: Linear correlation of LHCSR3 content per chlorophyll and maximum NPQ induction. In the graph the maximum NPQ measured for WT, *npq4 lhcsr1* and transformed lines cultivated in low light (LL) or high light (HL) is reported as a function of LHCSR3 content per CP43 (Figure 2). Linear fit is reported on red. In the inset table the fitting parameter are indicated.

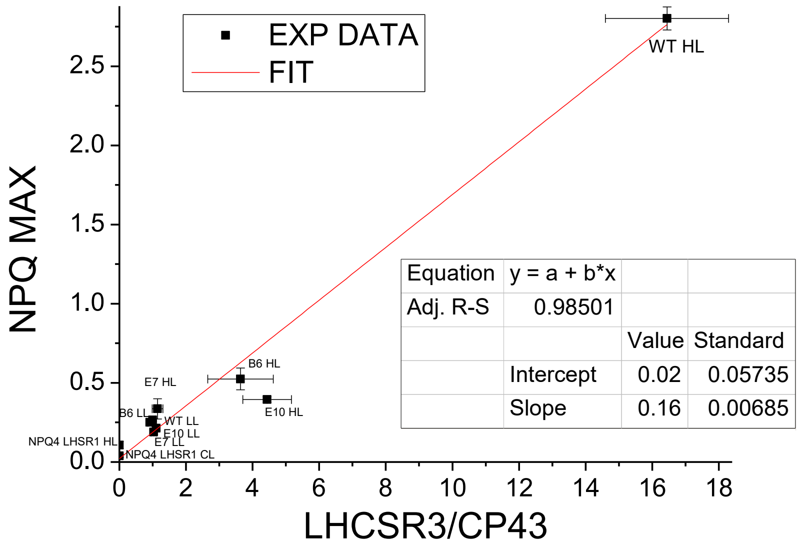

Figure S4: Immunoblot analysis of photosynthetic subunit accumulation normalized on a cell basis. Immunoblot signals reported in Figure 4 were analyzed by densitometry and reported normalized to the cell concentration. Standard deviation is indicated as error bars (n=4).

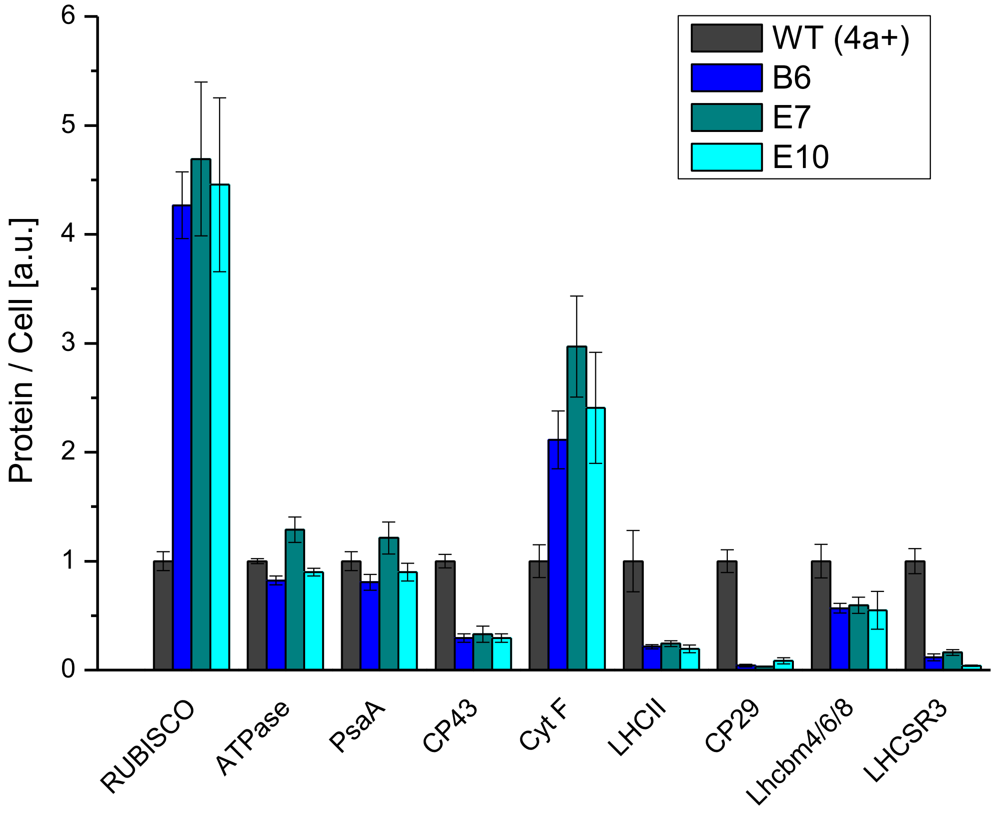

Figure S5: Immunoblot analysis of photosynthetic subunits accumulation in *npq4 lhcsr1* compared to WT. immunoblot analysis performed on WT and tranformed lines using specific antibodies for RuBisCO, ATPase β-subunit, CP43, PsaA, Cyt b6, LHCII, LHCBM6, and LHCSR3. Immunoblotting results were analysed by densitometry in order to determine the relative protein abundance. Each protein level was normalized to the WT protein level.

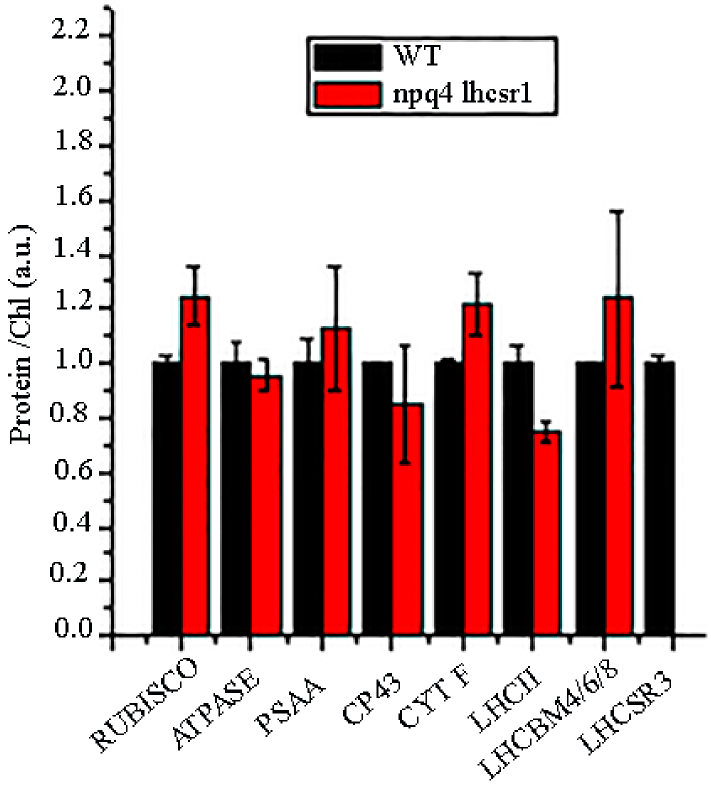

Supplement: Supplementary file 1 [file ijms-19-00155-s001.pdf]
